# Sugar-induced dysregulation of purine metabolism impacts lifespan

**DOI:** 10.18632/aging.104223

**Published:** 2020-12-27

**Authors:** Claudia Lennicke, Eliano dos Santos, Helena M. Cochemé

**Affiliations:** 1 MRC London Institute of Medical Sciences, Imperial College London, London, W12 8QA, UK

**Keywords:** sugar diet, metabolic disease, diabetes, obesity, purine catabolism, uric acid, *Drosophila*, aging

**Figure 1:**
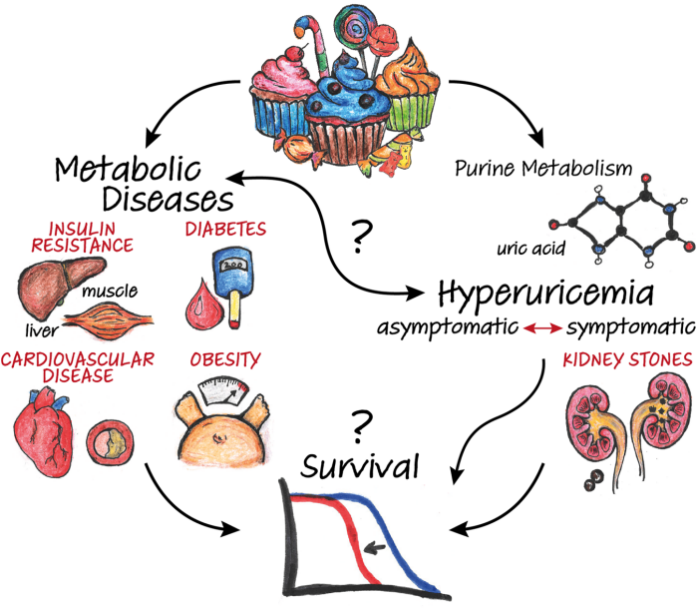
**Impact of diet-induced metabolic dysregulation on survival.** Excess dietary sugar leads to the dysregulation of carbohydrate and lipid metabolism, resulting in conditions such as obesity, insulin resistance, type-2 diabetes and cardiovascular disease. High-sugar diets also fuel purine catabolism, promoting the accumulation of uric acid as an end-product. Besides causing direct pathologies such as kidney stones, hyperuricemia (i.e. elevated uric acid levels) is associated with metabolic diseases and predictive of their onset. However, the underlying mechanisms and impact on survival are complex and remain to be fully elucidated.

Excess dietary sugar adversely affects human health by promoting the development of metabolic disorders such as obesity and type-2 diabetes (Figure 1). Sugar-rich diets are commonly used to replicate and investigate metabolic diseases in animal models, including the fruit fly *Drosophila melanogaster* [1]. High-sugar diets also shorten survival, which was widely attributed to these diabetic-like metabolic defects. However, our recent work in *Drosophila* reveals that longevity can be uncoupled from obesity and insulin resistance [2].

Instead, we show that a chronic high-sugar diet induces water imbalance in the fly, leading to thirst and dehydration, and that their decreased lifespan can be fully rescued by providing an *ad libitum* water source. Importantly, while longevity is restored to control levels, water supplementation does not rescue the metabolic complications typically associated with high-sugar diets, namely the accumulation of triacylglycerides, hyperglycemia, insulin resistance, and glycation damage. This indicates that these metabolic defects are not the limiting factor for fly survival *per se*.

As an alternative hypothesis, we propose that the pathophysiology of high-sugar diets is linked to the dysregulation of purine metabolism [3]. Prompted by the water effect, we focused on the fly tubules, which are functionally equivalent to the tubular portion of the mammalian kidney. Just like humans, the fly excretory system can develop lithiasis under dietary challenge [4]. We found that chronic high-sugar feeding fuels the accumulation of uric acid as a breakdown product of purine degradation, and induces the formation of uric acid stones in the fly tubules. Water supplementation can rescue this tubular dysfunction associated with excess dietary sugar, by reversing the dehydration and consequently diluting the concentration of stone-precursor metabolites.

Similarly, supplementing the control diet with excess purines leads to increased systemic levels of uric acid, and recapitulates the tubular pathology and shortened lifespan found on the high-sugar diet, which are also prevented by providing water. This suggests that water imbalance is the key driving factor in determining the longevity of flies under these challenging diets. The usual source of water in insect studies is the food itself, which is typically an agar-set medium containing sugar and yeast. Our results suggest that future studies, particularly those that rely on dietary manipulation to model diseases and those aimed at understanding health through longevity, should consider including a control group provided with *ad libitum* water to exclude a potential contribution of water imbalance in their interventions.

To further explore the importance of uric acid accumulation in stone formation and in determining fly survival independently of water balance, we treated flies on a high-sugar diet with the xanthine oxidase inhibitor allopurinol, a uric acid-lowering drug commonly prescribed in the clinic. Allopurinol prevents the formation of sugar-induced tubule stones and partially rescues the shortened lifespan of flies on a high-sugar diet. However, allopurinol treatment decreases fly survival on the control diet, associated with adverse side-effects of blocking a major cellular pathway such as purine degradation, and the deleterious accumulation of upstream metabolites. This suggests that allopurinol may not be an adequate pharmacological strategy for preventing diseases and improving survival associated with dysregulation of purine metabolism in a healthy population.

To assess whether dietary sugar intake also impacts purine metabolism in humans, we correlated dietary records with serum metabolomics data from a healthy human cohort of 650 participants. We found that consumption of sugar-rich foods indeed displays a strong association with renal dysfunction and elevated levels of circulating purines. This is consistent with our observations in the fly model, and suggest that dietary sugars may also affect human health via dysregulation of purine metabolism.

Further research is needed to understand whether a sugar-restricted diet may be an effective strategy to prevent uric acid-related diseases, and how purine metabolism modulates health beyond the disorders directly caused by uric acid crystallization, such as urolithiasis or gout, a form of inflammatory arthritis. Interestingly, systemic uric acid levels increase with age, and are linked to the incidence of metabolic disease and survival in humans. In prospective studies, asymptomatic hyperuricemia can predict the onset of conditions such as metabolic syndrome and type-2 diabetes [5,6], and is a risk factor for cardiovascular and all-cause mortality [7]. Therefore, understanding the pathophysiological links, especially whether these interactions are causal or correlational, will be critical for gaining mechanistic insight into how dysregulation of purine metabolism contributes to disease progression and survival. Overall, these investigations will support the discovery of new translatable targets and preventive strategies for healthier aging.
